# Understanding the Engagement and Interaction of Superusers and Regular Users in UK Respiratory Online Health Communities: Deep Learning–Based Sentiment Analysis

**DOI:** 10.2196/56038

**Published:** 2025-02-13

**Authors:** Xiancheng Li, Emanuela Vaghi, Gabriella Pasi, Neil S Coulson, Anna De Simoni, Marco Viviani, AD HOC Group

**Affiliations:** 1 School of Business and Management Queen Mary University of London London United Kingdom; 2 Department of Informatics, Systems and Communication University of Milano-Bicocca Milan Italy; 3 School of Medicine University of Nottingham Nottingham United Kingdom; 4 Wolfson Institute of Population Health Asthma UK Centre for Applied Research Queen Mary University of London London United Kingdom; 5 See Acknowledgments

**Keywords:** social media, online health communities, social network analysis, sentiment analysis, bio-bidirectional encoder representations from transformers, asthma, chronic obstructive pulmonary disease

## Abstract

**Background:**

Online health communities (OHCs) enable people with long-term conditions (LTCs) to exchange peer self-management experiential information, advice, and support. Engagement of “superusers,” that is, highly active users, plays a key role in holding together the community and ensuring an effective exchange of support and information. Further studies are needed to explore regular users’ interactions with superusers, their sentiments during interactions, and their ultimate impact on the self-management of LTCs.

**Objective:**

This study aims to gain a better understanding of sentiment distribution and the dynamic of sentiment of posts from 2 respiratory OHCs, focusing on regular users’ interaction with superusers.

**Methods:**

We conducted sentiment analysis on anonymized data from 2 UK respiratory OHCs hosted by Asthma UK (AUK), and the British Lung Foundation (BLF) charities between 2006-2016 and 2012-2016, respectively, using the Bio-Bidirectional Encoder Representation from Transformers (BioBERT), a pretrained language representation model. Given the scarcity of health-related labeled datasets, BioBERT was fine-tuned on the COVID-19 Twitter Dataset. Positive, neutral, and negative sentiments were categorized as 1, 0, and –1, respectively. The average sentiment of aggregated posts by regular users and superusers was then calculated. Superusers were identified based on a definition already used in our previous work (ie, “the 1% users with the largest number of posts over the observation period”) and VoteRank, (ie, users with the best spreading ability). Sentiment analyses of posts by superusers defined with both approaches were conducted for correlation.

**Results:**

The fine-tuned BioBERT model achieved an accuracy of 0.96. The sentiment of posts was predominantly positive (60% and 65% of overall posts in AUK and BLF, respectively), remaining stable over the years. Furthermore, there was a tendency for sentiment to become more positive over time. Overall, superusers tended to write shorter posts characterized by positive sentiment (63% and 67% of all posts in AUK and BLF, respectively). Superusers defined by posting activity or VoteRank largely overlapped (61% in AUK and 79% in BLF), showing that users who posted the most were also spreaders. Threads initiated by superusers typically encouraged regular users to reply with positive sentiments. Superusers tended to write positive replies in threads started by regular users whatever the type of sentiment of the starting post (ie, positive, neutral, or negative), compared to the replies by other regular users (62%, 51%, 61% versus 55%, 45%, 50% in AUK; 71%, 62%, 64% versus 65%, 56%, 57% in BLF, respectively; *P*<.001, except for neutral sentiment in AUK, where *P*=.36).

**Conclusions:**

Network and sentiment analyses provide insight into the key sustaining role of superusers in respiratory OHCs, showing they tend to write and trigger regular users’ posts characterized by positive sentiment.

## Introduction

### Background

Online health communities (OHCs) have been increasingly explored in recent years as a means of enabling people with long-term conditions (LTCs) to exchange peer self-management support [1-3]. Such communities offer an easily accessible and cost-effective means of sharing experiences, exchanging information, and providing mutual support to one another [4,5]. Participation in OHCs for individuals living with LTCs could address part of the health care service demand and indirectly improve access to health care [6]. The analysis of the role of OHCs in health promotion and management of LTCs indicates that there might be a positive effect on patients’ perception of social support, health literacy, clinical outcomes, and behavior change [7,8]. Furthermore, the involvement of patients in these OHCs can improve their engagement with respect to their care and their ability to self-manage, their mental health outcomes [9], and contribute to health equity [3]. However, despite the growing popularity of OHCs, there is still much we do not know about how these communities function [10]. Moreover, the specific nature of regular users’ interaction with the so-called superusers—that is, individuals who frequently engage with the community—and the extent to which it supports self-management remains largely unknown [7].

Recent social network analysis performed on 2 active respiratory OHCs has suggested that superusers play a critical role in holding together the community and ensuring timely exchange of support and information [5,10]. These superusers have been shown to contribute more content to the community, initiate more interactions, and respond more often to other users’ queries than regular members [8,9]. From a topological point of view, their characteristics are similar to those of hubs*,* that is, nodes with a disproportionally large number of connections compared to other nodes in the network.

Across a variety of empirical domains, it has been documented that hubs are valuable resources that help facilitate the spread of information widely and amplify information cascades [11], for example, help design effective vaccination campaigns and selective immunization strategies against disease diffusion and epidemics [12,13] and help improve the system’s robustness and vulnerability to random failures [14]. However, some hub identification approaches can be very time-consuming and suffer from the possibility that spreaders are so close together that they overlap the sphere of influence. In this context, VoteRank is a simple iterative method to identify a set of decentralized spreaders with the best spreading ability [15]. In this approach, all nodes vote in a spreader in each turn, and the voting ability of neighbors of the elected spreader will be decreased in the subsequent turn. It is, therefore, an effective solution for identifying possible nonoverlapping superusers.

However, the analysis of the network topology alone is not sufficient to fully understand the interactions between regular users and superusers and their impact on the whole community. For this reason, it is necessary to analyze the content of posts and what relationships (if any) exist between the 2 groups with respect to how they react to each other’s content. Sentiment analysis (SA), that is, a subfield of natural language processing provides an understanding of the sentiment of posts and whether there is a cause-and-effect relationship between posts in a thread. This approach consists of analyzing digital text to determine its polarity, that is if the emotional tone of the message is positive, negative, or neutral. SA can create structured and actionable knowledge from unstructured text for decision makers [16] in different fields, from marketing to politics and health [17]. In particular, with respect to the health domain, a variety of works have used SA techniques (both lexicon-based and semantic-based) [18] in recent years for different health conditions, for example, assessing the degree of psychological distress linked to COVID-19 [19,20], evaluate the risk of alcoholism in particular categories of users [21], analyze the emotional state of users with diabetes [22], or the role digital platforms in mediating health-related support with respect to specific cancer drugs [23]. In most of these works, a distinction is not made between categories of users, their interactions, and their role in OHCs.

This work is part of a research program that will eventually test whether promoting engagement in OHC improves self-management and clinical outcomes [24]. The primary motivation of this study is to investigate the engagement patterns of different user types, particularly superusers and regular users, and how their interactions influence the overall sentiment of posts. Our hypothesis is that superusers play a pivotal role in community cohesion, offering immediate access to a support network for self-management, as well as emotional and illness-related support. By doing so they foster positive sentiment among regular users, which subsequently may mediate improvements in self-management behaviors [25,26]. By understanding these dynamics, we aim to provide insights that can enhance the effectiveness of OHCs.

Using a semantic approach, this study aims to explore the sentiment of posts in 2 dynamic and active respiratory OHCs; in doing this, regular user interactions with superusers are assessed, in order to shed light on the impact of such interactions on users’ sentiment and which may ultimately impact on the self-management of their LTCs. Specifically, we investigate the sentiments of both regular users and superusers expressed in these interactions as well as their patterns over time. Additionally, we aim to compare the sentiment of superusers’ posts, with superusers defined in 2 different ways, one with emphasis on high-posting activity and the other on high-spreading ability, to verify whether they display similar characteristics or represent indeed the same population.

By shedding light on these critical aspects, this study contributes to understanding the mechanisms underlying the effectiveness of OHCs as a tool to facilitate self-management and provides insights into how respiratory OHCs may meet the needs of their users.

### Data

As described in our previous work [10], data were collected by HealthUnlocked [27], the platform provider of the Asthma UK (AUK) and British Lung Foundation (BLF) communities. In both communities, registered users can choose to either write posts publicly or send private posts to one another. In the latter case, posts are shared between 2 users only, whereas when posts are written publicly, other users can become connected through threads of posts. For this study, only posts that were shared publicly were considered. Our datasets were stored and analyzed in a Safe Haven space, that is, a secured database held by Queen Mary University. Anonymized user IDs were provided by HealthUnlocked, and no demographic information was available. The datasets included posts and their metadata including the date of posting, the hierarchical level of the post within the corresponding thread, and the dates in which the users joined and left the community. No data were collected on participants’ characteristics, though only people declaring themselves to be older than 16 years of age were permitted to create an account and take part in OHCs.

Six different types of data associated with the corresponding user actions were collected for each user including (1) posts followed, (2) users followed, (3) likes, (4) level-0 posts (ie, posts starting new threads), (5) level-1 replies (ie, replies to the level-0 posts), and (6) level-2+ replies (ie, replies to other replies beyond level 2). The original datasets consisted of 32,780 data items associated with 3345 users from 2006 to 2016 for AUK, and 875,151 data items associated with 19,837 users from 2012 to 2016 for BLF. Since in this study, we are interested in analyzing only the textual content associated with posts, and some of them turned out to be without any content, they were removed from the datasets. The final datasets, therefore, contained 12,413 and 369,224 posts for AUK and BLF, respectively. In 2015, HealthUnlocked took over the AUK forum, leading to substantial increases in posting activity and volume of users. Further details are provided in the posting activity section of our previous work [10].

## Methods

### Study Design

Superusers were first identified using two different methodological approaches: (1) their posting activity and (2) their spreading ability. Next, SA was applied first to all posts and then to interactions between superusers and regular users.

### Identification of Superusers

Two ways of identifying superusers were considered. The first method was based on identifying the “top 1% of users characterized by the largest number of posts written in the community over the entire observation period,” as previously described in a study by Joglekar et al [10]. The second method approximates being a “spreader” to being a superuser, according to the VoteRank algorithm [15]. This algorithm is implemented in Python (Python Software Foundation) in the *NewtworkX* package [28]. The VoteRank algorithm finds the top-ranked nodes as spreaders according to an influence ranking. The idea behind VoteRank’s rank is to choose a set of spreaders one by one according to the voting scores of nodes obtained from the neighbors. The node that gets the most votes in each turn is selected as the most influential node. It is an iterative method where at the beginning all nodes take part in ranking their neighbors. However, when a node is identified as a spreader, it will no longer take part in subsequent iterations and neighboring nodes will have a penalty, so that nodes that are not significant in the transmission of information but exploit proximity to the influencing nodes are not considered as spreaders. To make a fair comparison, we identified and compared the same number of superusers according to the 2 definitions. This was achieved by picking the *k* top-ranked spreaders by VoteRank, where *k* is the number of superusers according to the “top 1%” definition.

### Sentiment Analysis

SA was carried out by means of a semantic approach based on bidirectional encoder representations from transformers (BERT). BERT is a contextualized word representation pretrained language model [29,30]. Its architecture is a multilayer bidirectional transformer encoder based on the original transformer implementation. Vaswani et al [31] shows further details about the transformer architecture.

BERT is built in 2 steps: pretraining and fine-tuning. The model is trained first on unlabeled data over different pretraining tasks. Later, the model with the pretrained parameters is fine-tuned using labeled data from downstream tasks. Every task has a separate fine-tuned model. However, a unique characteristic of BERT is that it has a unified architecture across different tasks, so the difference between pretrained architecture and the final downstream is small. We used BioBERT (Bio-Bidirectional Encoder Representation from Transformers), which is a pretrained language representation model for the biomedical domain [32].

### Fine-Tuning BioBERT for SA

BioBERT was fine-tuned using a COVID-19 Twitter Dataset [33] taken from Kaggle [34]. The choice was opportunistic as there were few open datasets related to health. The COVID-19 Twitter Dataset has a total of 143,903 usable records, with labels associated with neutral, positive, and negative posts. Numeric values have then been associated with sentiment: 0 for neutral, 1 for positive, and –1 for negative posts. Examples of posts with sentiment labels are given in the Multimedia Appendix 1. Normalization and stop-words removal were performed on both datasets.

**Figure 1:**
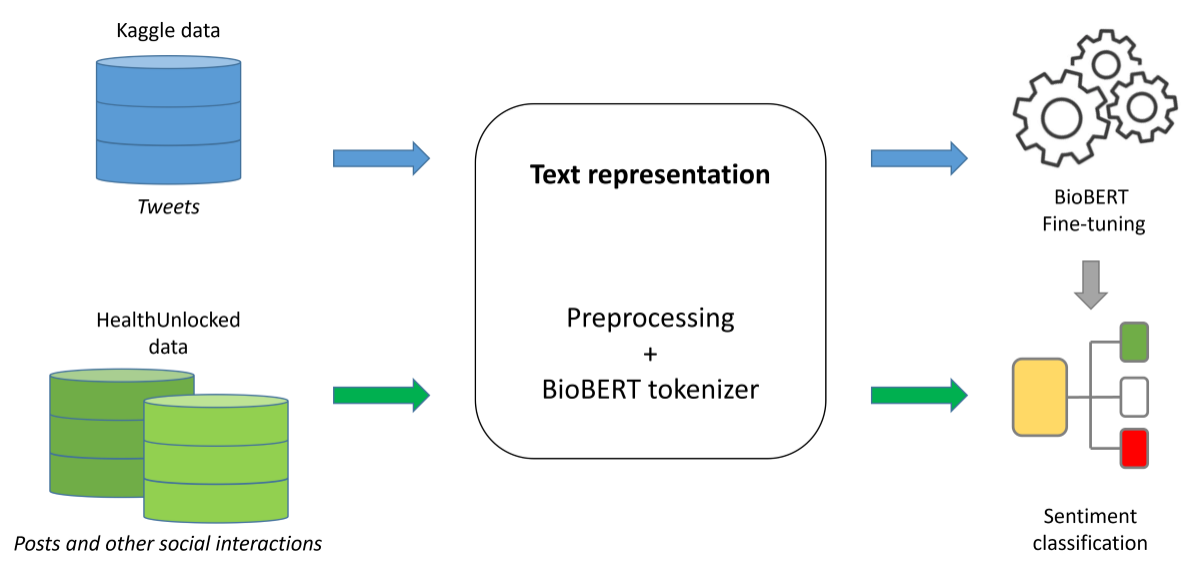
Sentiment analysis workflow. BioBert: Bio-Bidirectional Encoder Representation from Transformers.

The considered SA workflow performed on AUK and BLF is illustrated in Figure 1. The BioBERT model was initialized by using the standard configurations and weights from the Hugging Face repository [35]. As a first step, we converted the data to sequences, adding the tags to indicate where the sentence starts and its separator. Then, we tokenized the resulting sequences with the BioBERT tokenizer creating a tensor dataframe of 128 characters. The dataset was then split into training and validation sets, with 85% observations for training and 15% observations for validation. The Adam function was used as an optimizer [36], the sparse categorical cross-entropy as loss function [37], and the sparse categorical accuracy as a metric for training [38]. The training was performed on just 2 epochs with a batch size of 32. Google Colab Pro was used to fine-tune BioBERT [32]. After the fine-tuning, weights, and result configuration files were stored.

### SA on AUK and BLF

By using the earlier fine-tuned model, we performed SA on AUK and BLF data, including all posts (it is important to note that in this paper when we talk about posts, we also generally include replies; when we consider only replies, we refer to them directly). We performed the following analyses.

#### Average Sentiment

Average sentiment scores (AVSs) for both regular users’ and superusers’ posts have been computed separately. As sentiment labels are associated with numeric values (ie, 0 to neutral, 1 to positive, and –1 to negative), the average values of the sentiment of a set of posts can be computed and used to capture the general sentiment expressed in those posts. Specifically, the AVSs range in the [–1*,* 1] interval, where positive values and getting closer to 1 indicate increasing positive sentiment, while negative values and getting closer to –1 indicate increasing negative sentiment. We considered different aggregations of content types as follows: (1) 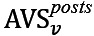 , the AVS of all posts of a set of users in *v*; that is, either regular users (*U*) or superusers (*S*); (2) 
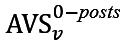
, the AVS of level-0 posts of users in *v*; (3) 
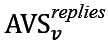
, the AVS of all replies given by users in *v*; (4) 
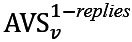
, the AVS of level-1 replies given by users in *v*; and (5) 
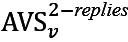
, the AVS of level-2+ replies given by users in *v*.

Along with the AVSs, we also computed the percentage of posts with different sentiments to show the distribution of sentiment expressed in users’ posts. To investigate the trend of the average sentiment over time, we sorted all posts based on their publication time and regrouped them into 15 bins with an equal volume, for which we computed AVSs. We did not aggregate posts based on the month and year of publication because the number of available data was too small in AUK before 2015 (when HealthUnlocked took over the forum), and because the period of analysis in the 2 communities was different. 2-tailed *t* tests were used for statistical significance when comparing 2 AVSs of different users.

#### User-Superuser Interaction Sentiment

These analyses investigated the interactions between regular users and superusers. We addressed the following.

- Regular users’ and superusers’ sentiments when replying to each other: To do this, we first identified threads started by regular users and superusers, denoted as TU and TS, and calculated the AVS of replies written by the other group of users. Specifically, we computed 
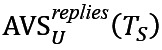
, which denotes the average sentiment of regular users in reply to superusers' initiated threads; and 
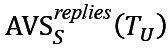
, which denotes the average sentiment of superusers in reply to regular users' initiated threads.
- Regular users’ sentiments when replying to other regular users and superusers: Here, we focused on regular users’ replies only and checked whether they acted differently when replying to other regular users or superusers. Specifically, we compared 
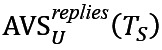
 with respect to 
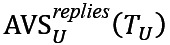
 which denotes the average sentiment of regular users in replying to other regular users' threads. A similar approach to that introduced earlier was used to analyze the trend in the value of 
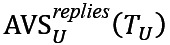
 over time.
- Superusers’ sentiments when replying to regular users: In this case, we only took into consideration the sentiment of the replies superusers give to regular users. Note that superusers also interact with each other, but the study of these interactions is beyond our research questions and is not shown here. We investigate the sentiment of superusers in reply to positive, negative, and neutral level-0 posts of regular users. To do this, we computed 
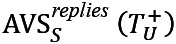
, which denotes the average sentiment of superusers in replying to regular users’ positive level-0 posts; 
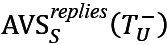
, which denotes the average sentiment of superusers in replying to regular users' negative level-0 posts; and 
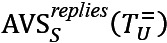
, which denotes the average sentiment of superusers in replying to regular users’ neutral level-0 posts.

Regular users’ replies to other regular users’ level-0 posts are used as a baseline and compared with AVSs of superusers. This analysis assesses in more detail the superusers’ tendency to act as help-givers [10], especially when regular users express negative sentiments. Along with the AVSs, we also computed the percentages of posts with different sentiments to show the distribution of sentiment expressed in users’ posts. The 2-sample *z* tests for proportions were used for statistical significance when comparing 2 percentages of different users.

A similar approach to that introduced earlier was used to analyze the trend in these percentages over time.

### Ethical Considerations

The study was approved by the Queen Mary University Research Ethics Committee (QMERC 22.279). The research team did not have access to personally identifiable information. The data was anonymized to ensure user privacy, and no demographic information was included in the analysis. The posts analyzed were publicly available, with the users having consented to their use for analytical purposes by choosing to share them publicly. In addition, the research protocol was examined and permission to undertake the research was obtained by AUK and BLF charities, and as well as HealthUnlocked. To further protect the privacy of the users, no posts are directly quoted.

## Results

### Overview

**Figure 2:**
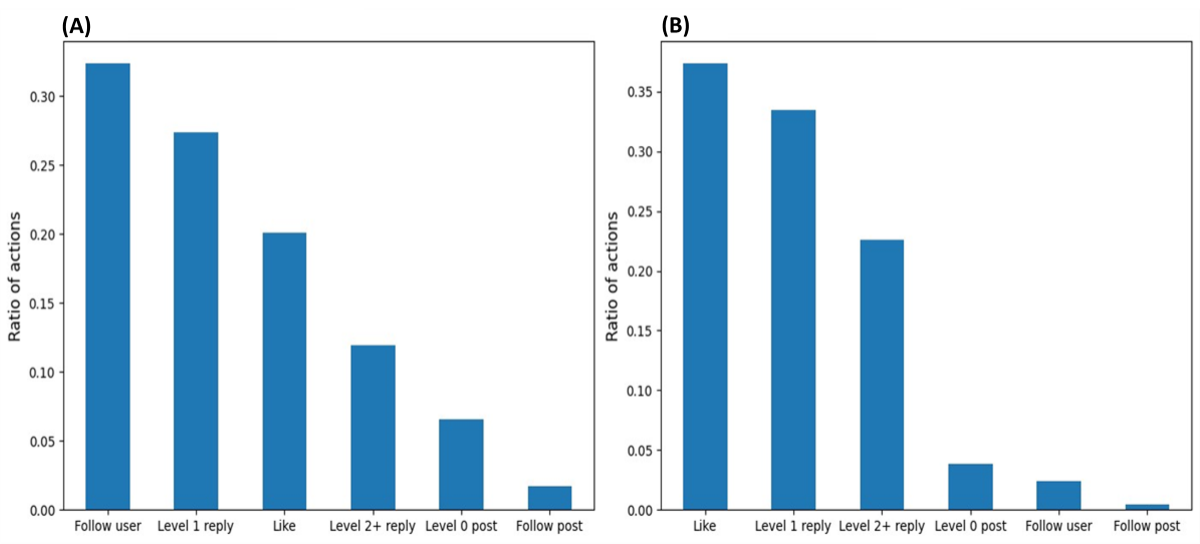
Distribution of different actions in distinct communities. (A) Asthma UK (AUK); (B) British Lung Foundation (BLF).

The ratio of distinct actions over all actions performed with respect to both OHCs is shown in Figure 2. In AUK, the most common action was to “follow” users, while for BLF was to “like” posts or replies. In both communities, the action of generating level-0 posts, that is, starting a new thread, was smaller than generating both level-1 and level-2+ replies, showing that users mostly communicated through replies.

### Identification of Superusers

Using the definition of superusers as the “top 1% of users characterized by the largest number of posts written in the community over the entire observation period” yielded 33 and 198 superusers in the AUK and BLF communities, respectively. A detailed description of users’ posting activity can be found in our previous work [10].

Then, we used the second definition of superusers with VoteRank to identify the same numbers of superusers. The ratio of overlap of superusers according to the 2 definitions was greater than 60% (20/33 in AUK and 156/198 in BLF), showing that users who posted the most (1% definition) were largely overlapping with the spreaders (VoteRank definition). As the results observed from both definitions were consistent in this study, we only show in the following the results obtained by using the 1% definition and have moved those obtained with the VoteRank definition to Multimedia Appendix 2. In both communities, 60%-70% of registered users never wrote any posts, which shows that the majority of regular users were passive (ie, the so-called lurkers).

**Figure 3:**
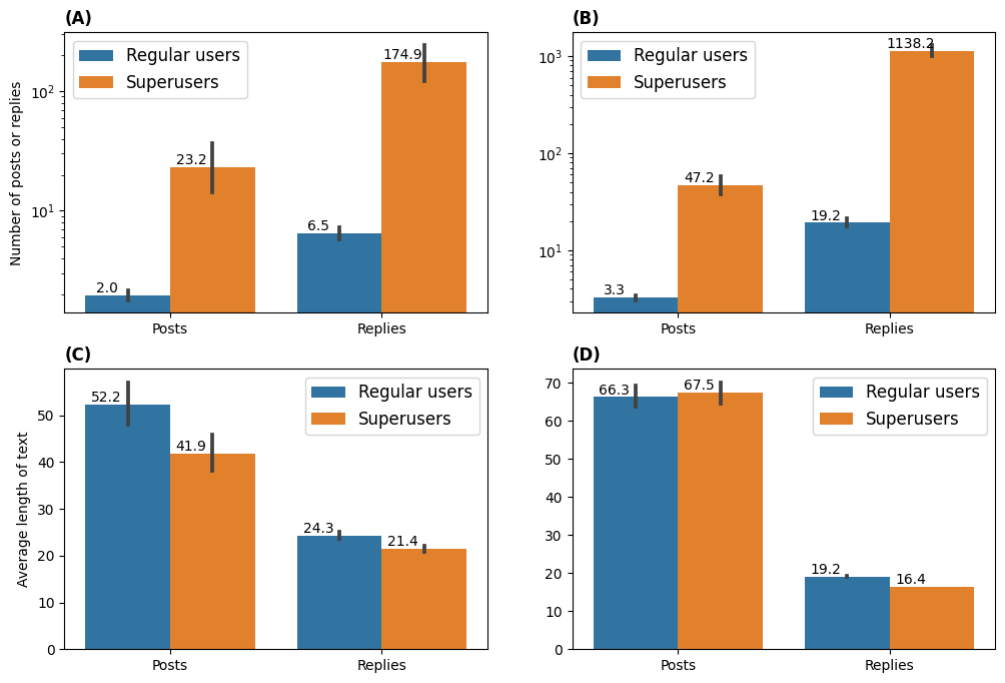
Comparisons of the number and length of posts written by superusers and regular users. (A,C) Asthma UK (AUK); (B,D) British Lung Foundation (BLF).

Figure 3 compares the number of posts as well as the length of text written by both categories of users. Specifically, Figures 3A and 3B show that superusers tended to reply more often than starting new posts, as described in our previous work [10]. Figures 3C and 3D show that both superusers and regular users tended to write longer level-0 posts than replies. In addition, we can observe that superusers’ level-0 posts and replies are significantly shorter than regular users’ ones in AUK, while they are of similar length in BLF.

### Sentiment Analysis

The fine-tuned BioBERT-based SA with the COVID-19 Twitter Dataset achieved an accuracy of 96% on the validation set.

### Average Sentiment

**Figure 4:**
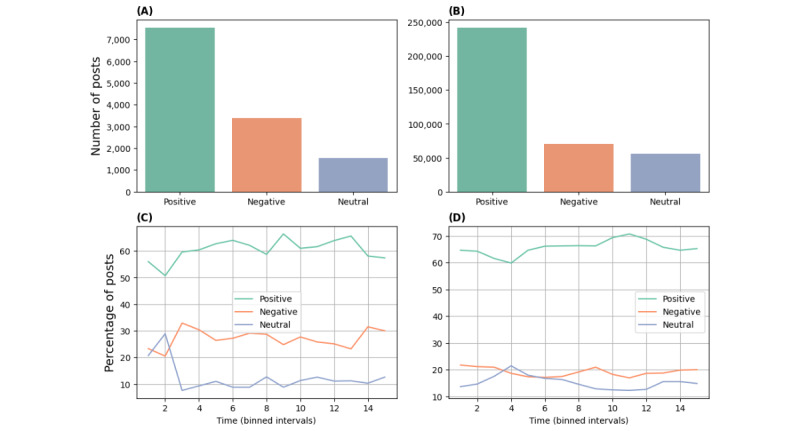
Number of posts and percentage of posts with different sentiments over time. (A,C) Asthma UK (AUK); (B,D) British Lung Foundation (BLF).

In both communities, most posts were associated with positive sentiment, as illustrated in Figure 4. Figures 4C and 4D show the percentage of posts with positive, negative, and neutral sentiments, across the 15 bins, related in Figures 4A and 4B to sentiment versus total number of posts. In both communities, posts with positive sentiment were always the ones with higher frequency.

Table 1 shows the percentages of sentiment across all posts, along with the overall average sentiment. The average sentiment of superusers’ posts was consistently higher than the one for regular users. Both superusers and regular users tended to be significantly more positive in replies compared to their level-0 posts (*P<.*001).

**Figure 5:**
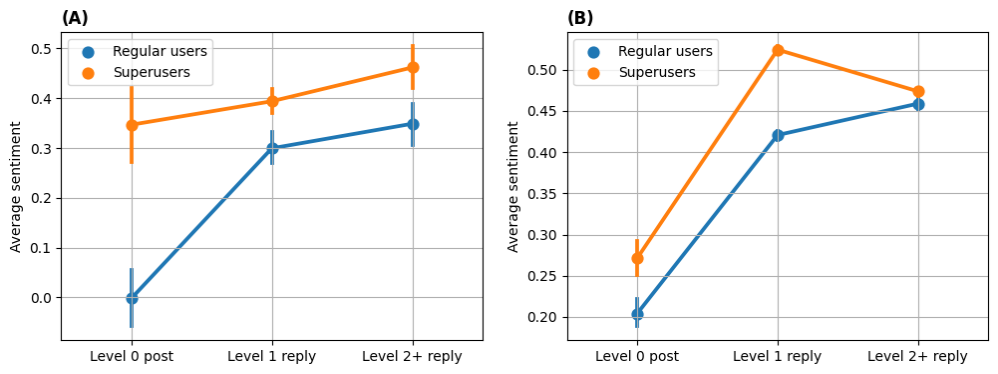
Average sentiment score (AVS) in different actions. (A) Asthma UK (AUK); (B) British Lung Foundation (BLF).

Figure 5 illustrates the average sentiment in level-0 posts, level-1 replies, and level-2+ replies. Although the patterns of superusers’ sentiment were different in the two communities, regular users tended to be the most positive in level-2+ replies and least positive in level-0 posts. This result further confirms that regular users tended to be more positive when having in-depth communications with others in the community.

**Figure 6:**
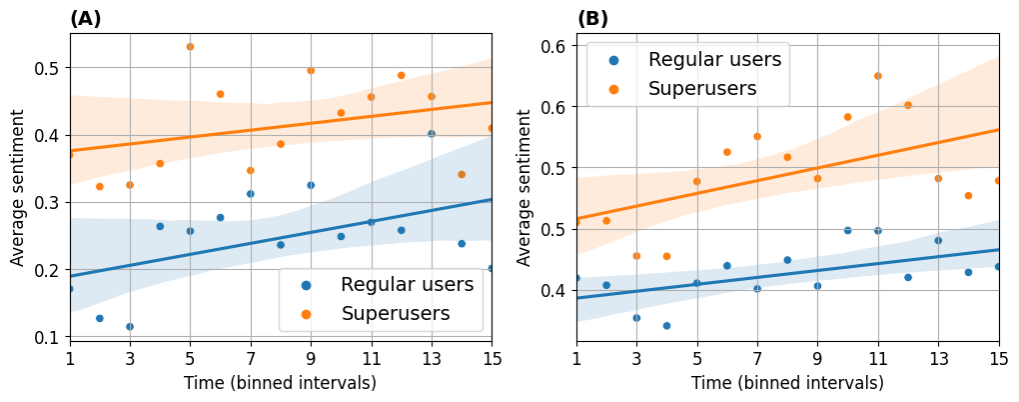
Trend of sentiment. All posts are sorted based on their publication time and regrouped into 15 bins with equal volume. Average sentiment of all posts written by regular users and superusers are calculated separately in each bin. (A) Asthma UK (AUK); (B) British Lung Foundation (BLF).

Figure 6 shows the trend of regular users’ and superusers’ sentiments over time. There is a trend toward positive sentiment for all users, with superusers being consistently more positive than regular users.

**Table 1:** Percentages of posts or replies with different sentiments and the average sentiments indicated as AVS^a^. Depending on the target, it refers to either 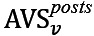, 
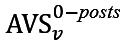
, or 
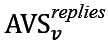
.

		AUK^b^		BLF^c^	
		Superusers	Regular users	Superusers	Regular users
**All** **posts**					
	Negative, %	22.94	31.46	17.20	22.34
	Neutral, %	13.64	11.03	15.92	14.03
	Positive, %	63.41	57.50	66.88	63.63
	AVS	0.405	0.26	0.497	0.413
**Level-0 posts**					
	Negative, %	30.19	45.92	30.92	33.86
	Neutral, %	4.95	8.35	11.06	11.88
	Positive, %	64.86	45.73	58.03	54.26
	AVS	0.347	-0.002	0.271	0.204
**Replies**					
	Negative, %	22.16	28.42	16.73	21.13
	Neutral, %	14.59	11.60	16.09	14.26
	Positive, %	63.25	59.98	67.19	64.62
	AVS	0.411	0.316	0.505	0.435

^a^AVS: average sentiment score.

^b^AUK: Asthma UK.

^c^BLF: British Lung Foundation.

### User-Superuser Interaction Sentiment

#### Regular Users’ and Superusers’ Sentiments When Replying to Each Other

We first analyzed how regular users reacted to superusers’ posts. We specifically identified the threads started by superusers and investigated the sentiments as well as the lengths of replies written by regular users in those threads (Table 2). We compared them with the replies written by superusers in threads started by regular users (Table 2). We showed the number of threads started by different groups of users, the number of threads with replies from the other group of users, AVS, and the average length of text (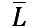). We calculated the AVS and 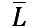 of (1) posts written by the focal group with replies from the other group, and (2) replies written by the other group of users.

We can observe that, in both communities, superusers started more than a third of all threads (ie, 626/1680 in the AUK and 7831/20,756 in the BLF communities). Regarding the sentiment in posts and replies, the average sentiment of starting posts written by superusers with replies written by superusers at the start of a thread was much more positive than those written by regular users (ie, 0.395 vs –0.004 in AUK and 0.25 vs 0.137 in BLF). Superusers tended to reply in a way that was much more positive than the sentiment expressed in the level-0 posts of regular users. Most interestingly, the sentiment of regular users in reply to superusers’ threads was as positive (0.489 vs 0.470 in BLF) or even more positive than superusers’ sentiment when replying to regular users (0.447 vs 0.352 in AUK).

In terms of the length of texts, similar to what we found in Figure 3, replies of both superusers and regular users tended to be shorter than level-0 posts. However, superusers tended to write longer texts when replying to regular users than regular users replying to them. This is opposite to what we found in Figure 3, where we show the overall length of superusers’ replies tended to be shorter than regular users. This observation suggests that superusers were willing to provide more information as help-givers when replying to regular users.

**Table 2:** Interactions between superusers and regular users. Depending on the target, the AVS^a^ of posts and AVS of replies refer to either 
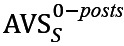
 and 
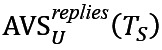
, or 
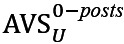
 and 
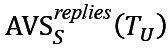
. Posts without any replies are excluded from the AVS calculation.

	Threads in AUK^b^			Threads in BLF^c^	
	Superusers	Regular users	Superusers		Regular users
Total threads	626	1054	7831		12,925
Threads with replies	352	719	6088		10,768
AVS of posts	0.395	–0.004	0.250		0.137
AVS of replies	0.447	0.352	0.489		0.470
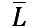 ^d^ of posts	36.764	47.250	68.836		42.487
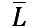 of replies	19.266	25.410	16.464		20.029

^a^AVS: average sentiment score.

^b^AUK: Asthma UK.

^c^BLF: British Lung Foundation.

^d^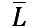: average length of text.

#### Regular Users’ Sentiments When Replying to Other Regular Users and Superusers

To explore how regular users reacted toward superusers’ threads, we investigated superusers’ posts with different sentiments separately. Table 3 represents the number of posts written by superusers, the percentage of posts with regular users’ replies, and the average sentiment and length of text in those replies. In both communities, the sentiment in regular users’ replies was largely affected by the sentiment of superusers in their posts. Regular users tended to be less positive and write longer texts when replying to superusers’ posts with negative sentiments.

**Figure 7:**
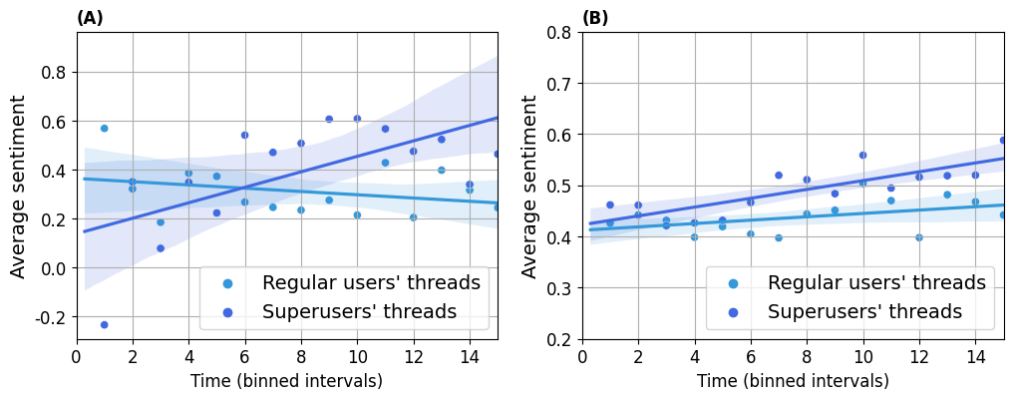
Sentiment trend in interactions between superusers and regular users. Average sentiment of regular users when replying to threads started by other regular users and superusers are calculated separately in each bin. (A) Regular users' sentiments Asthma UK (AUK); (B) Regular users' sentiments British Lung Foundation (BLF).

To investigate how regular users’ sentiments in replies to superusers’ posts change over time and whether they behaved differently when replying to superusers, we show the trend of regular users’ sentiments in threads started by superusers and regular users in Figure 7. In both communities, the sentiment of regular users when replying to superusers’ threads was becoming more positive while their sentiment in replying to regular users’ threads was relatively stable. The results suggest that superusers were somehow instrumental in the trend toward more positive sentiment from regular users over time.

**Table 3:** Summary of superusers’ threads and how regular users replied to them. 
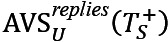
, 
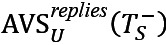
, and 
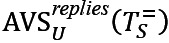
 are computed for AVS^a^ in corresponding columns.

	Sentiment of superusers (AUK^b^)			Sentiment of superusers (BLF^c^)		
	Negative	Neutral	Positive	Negative	Neutral	Positive
Posts	189	31	406	2421	866	4544
Replies, %	51.32	61.29	58.13	81.91	69.40	77.11
AVS of replies	0.230	0.508	0.495	0.364	0.435	0.503
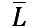 ^d^ of replies	24.11	19.41	19.14	19.84	14.11	16.76

^a^AVS: average sentiment score.

^b^AUK: Asthma UK.

^c^BLF: British Lung Foundation.

^d^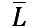: average length of text.

#### Superusers’ Sentiments When Replying to Regular Users

In Table 4, we compared the sentiments of superusers and regular users when replying to threads started by regular users with different sentiments expressed in their level-0 posts. From the sentiment in regular users’ replies, we found that regular users’ sentiment was largely affected by the sentiment expressed in the level-0 posts. On the other hand, superusers tended to write positive replies in threads started by regular users, whatever the type of sentiment of the starting post, positive, neutral, or negative, compared to regular users (62%, 51%, and 61% vs 55%, 45%, and 50% in AUK and 71%, 62%, and 64% vs 65%, 56%, and 57% in BLF, respectively). *P<*.001 for all pairs except for that in neutral threads in AUK, where *P*=.36. The result suggests superusers replied to regular users in a positive way as help-givers.

**Figure 8:**
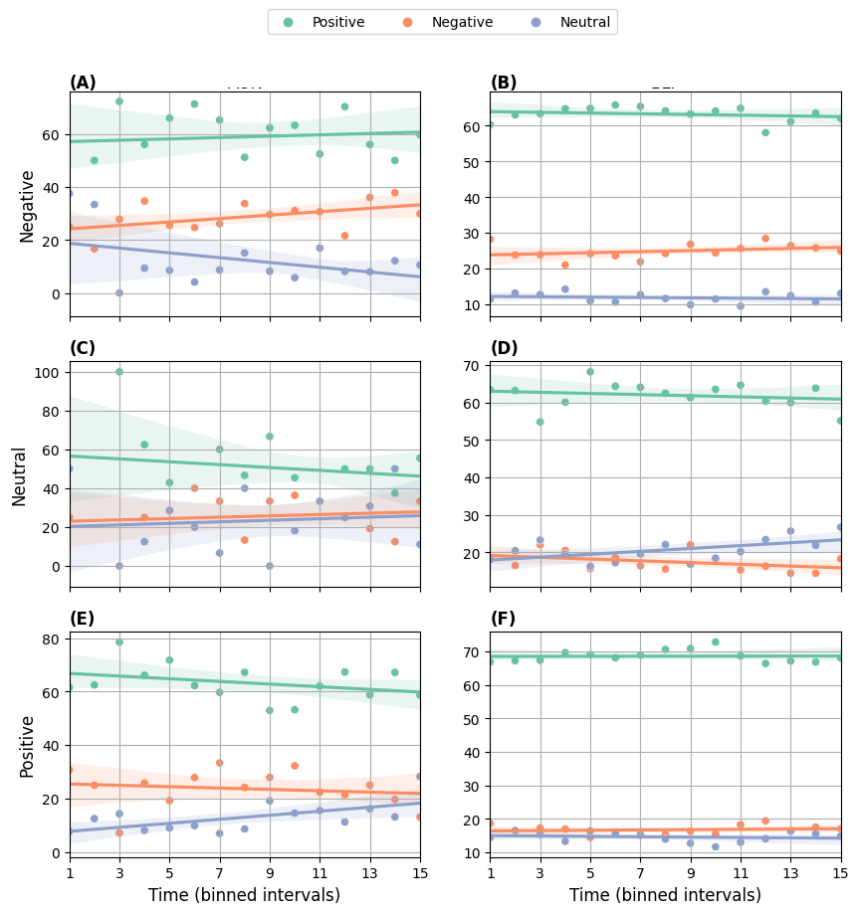
Sentiment trend in replies of superusers to regular users. Percentages of replies with corresponding sentiments are computed and shown in each bin. (A,C,E) Asthma UK (AUK); (B,D,F) British Lung Foundation (BLF).

In Figure 8, we show the percentage of superusers’ replies against the sentiments of the users’ posts with different sentiments over time. The percentage of superusers’ positive replies only marginally changed over time, and the majority of superusers’ replies were consistently associated with positive sentiments, whatever the users’ posts’ sentiment.

**Table 4:** Percentage of replies with different sentiments and average sentiments based on the sentiment in the level-0 post in each thread written by regular users. 
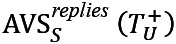
, 
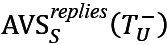
, and 
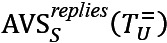
 are computed for AVS^a^ in corresponding columns. Regular users’ replies to other regular users’ level-0 posts are used as a baseline and compared with AVSs of superusers.

			Sentiment of regular users (AUK^b^)						Sentiment of regular users (BLF^c^)				
			Negative		Neutral		Positive		Negative		Neutral		Positive
**Regular users' replies**													
	Negative, %	39.83		21.15		31.93		31.03		22.58		20.80	
	Neutral, %	9.87		33.65		12.09		11.82		20.48		14.07	
	Positive, %	50.30		45.19		55.98		57.15		56.94		65.13	
	Total, %	100		100		100		100		100		100	
	AVS	0.105		0.24		0.24		0.261		0.344		0.443	
**Superusers' replies**													
	Negative, %	30.87		24.72		25.83		25.41		16.75		15.93	
	Neutral, %	7.83		23.60		11.75		10.07		20.36		12.75	
	Positive, %	61.30		51.69		62.42		64.53		62.89		71.32	
	Total, %	100		100		100		100		100		100	
	AVS	0.278		0.319		0.388		0.395		0.463		0.567	

^a^AVS: average sentiment score.

^b^AUK: Asthma UK.

^c^BLF: British Lung Foundation.

## Discussion

### Principal Results

This study shed novel light on regular users’ and superusers’ engagement in OHCs and on the impact of their interaction on the sentiment of communication (ie, posts). First, we showed that superusers who posted the most were largely overlapping with the “spreaders” of information and support. Then we found that superusers had a key role in promoting positive sentiment in OHCs, which could represent one of the mechanisms underlying their OHC “sustaining” role. In both OHCs, the great majority of posts were characterized by positive sentiment. This trend did not change over time. The average sentiment of superusers’ posts was consistently higher than that of regular users. Both superusers and regular users tended to be significantly more positive in their replies, especially at level 2+ replies compared to their sentiments in level-0 posts. Although this was likely due to the first posts of threads including a request for help presented within the context of a personal story, this could also be interpreted as regular users becoming more positive as a result of the engagement with the community and interaction with superusers. Indeed, the majority of superusers’ replies were consistently characterized by positive sentiments, whatever the users’ sentiment (negative, neutral, or positive) at the start of threads. Superusers wrote longer posts when replying to regular users, despite their overall tendency to write short posts. This suggests that superusers were willing to dedicate more time to providing information in replies to regular users compared to other regular users themselves. Moreover, we found evidence of regular users’ change toward a more positive sentiment after interaction with superusers. In fact, there was a trend toward more positive sentiment by regular users when replying to threads started by superusers, while their sentiment in replying to threads started by regular users was relatively stable. These results suggest that the active participation of superusers with consistently positive sentiment can not only enhance community cohesiveness but also foster an encouraging environment conducive to positive interactions and ensure the effective spread of informational and emotional support [4-9].

These findings have significant implications for both researchers and policy makers. Superusers in OHCs may represent a scalable and cost-effective health care workforce, providing a means for health and social care integration [6]. Their significant contributions have the potential to alleviate pressure on formal health services by promoting self-management and enhancing community-based care. As current health care challenges emphasize self-care and local service expansion [10], superusers could provide vital support by increasing the confidence of individuals in managing their conditions, reducing the demand on general practices, emergency care, and hospitals, and ultimately saving money within health care systems and across society.

By understanding the mechanisms of community sustainability and the impact of superuser involvement, policy makers and researchers can leverage these individuals as part of a broader health care team to develop and test more effective interventions. Future research is needed to explore how superusers might be formally recognized as contributors to health care, functioning as allied professionals within digital communities. This approach addresses the need for accessible and effective self-management interventions that integrate peer support into health care systems.

### Strengths and Limitations

A key strength of this work is the use of previously characterized datasets from 2 established active and dynamic respiratory OHCs [10]. The paper analyzes extensive data from the 2 significant OHCs with activities of more than 20,000 users in total. Based on SA, this work has started elucidating crucial mechanisms underlying the potential of superusers to affect the sentiment of OHC users. Besides, the paper shows the consistency in results with 2 different definitions of superusers, providing a robust basis for its findings.

A limitation of this study is the lack of a domain-specific labeled dataset for the fine-tuning of BioBERT. The choice to use this dataset for fine-tuning BioBERT was opportunistic as there are few open datasets related to health in social networks, and most are about COVID-19. In our study, BioBERT was fine-tuned on a COVID-19 Twitter Dataset. The brevity of Twitter posts, constrained by character limits, might restrict the richness of health discussions compared to longer, more detailed posts found in OHCs. Additionally, the intense emotional tone surrounding the COVID-19 pandemic may skew SA results toward more extreme expressions of sentiment. This could result in a bias toward stronger sentiment expressions in our model’s predictions, potentially differing from sentiment trends in other health-related datasets that are not solely concerned about global health crises. A further limitation is the lack of demographic and clinical information of participants as well as verification and validation of the information shared in the OHCs. In this study, the contents of some posts were analyzed for the validity of our sentiment model, however, findings were not validated through a comprehensive semantic analysis of most posts.

### Comparison With Prior Work

The results of this study are consistent with previous work and help illuminate the dynamics within OHCs [10]. These results are also in keeping with previous research on emotional contagion [29], which has shown that one person’s mood might fleetingly determine the mood of others. Previous work in social networks suggests that happiness is a network phenomenon, clustering in groups of people that extend up to three degrees of separation [30]. Methodologically, users’ sentiment was extracted with semantic-based techniques, which is a common approach used in previous research [19-21]. In most of these works, a distinction is not made between categories of users, their interactions, and their role in OHCs. By investigating the sentiments of regular users and superusers as well as during their interactions, this study provides novel insights into the essential role played by superusers. In agreement with previous research, superusers tended to reply to other users’ posts [10] with concise posts in general. In addition, when replying to regular users, they tended to provide more information by writing longer texts. Besides, they contribute more content to the community and respond more often to other users’ queries than regular members [8,9]. Comprehending the dynamics of superuser interactions can help us understand more about what effective peer moderation could look like (ie, moderation by superusers). It also helps design strategies for better moderation and engagement in OHCs, potentially improving the support and information exchange in these communities.

### Conclusions and Future Research

SA provides insight into the key sustaining role of superusers in respiratory OHCs, showing they tend to write and respond with posts with positive sentiment. Future research is needed to develop an approach able to understand the sequence of responses in a discussion thread within the OHC datasets to understand whether a superuser’s response was successful in changing the user’s sentiment in the case of negativity. Moreover, the existence of groups of users writing posts with negative sentiments could be explored, whether these users form “echo chambers” [39], therefore reinforcing beliefs and reducing exposure to users with alternative views on the topic of discussion. The evolution of such echo chambers over time could also be characterized. Another interesting development could be topic modeling analysis, in order to test the existence of subcommunities based on topics of discussion, but also to investigate the correlation between topics and sentiment types, and user characteristics associated with different sentiments concerning different topics. In addition, it would be interesting to analyze sentiment associated with topics of discussion, for example, symptoms and treatment, to inform the moderation process and provide tailored information or support. The ultimate aim would be to develop strategies for better OHC moderation, enhanced effectiveness of engagement, and improved OHC users’ safety so that engagement in OHCs could be integrated within health care services for patients with chronic conditions.

## Appendix

Multimedia Appendix 1 Examples of random posts with different sentiment classified by BioBERT.

Multimedia Appendix 2 Results with superusers identified by VoteRank.

## Abbreviations

AUK: Asthma UK
AVS: average sentiment score
BERT: bidirectional encoder representations from transformers
BioBERT: bio-bidirectional encoder representation from transformers
BLF: British Lung Foundation
LTC: long-term conditions
OHC: online health community
SA: sentiment analysis

## Data Availability

Data sharing is not applicable to this article as no datasets were generated during this study.
