# Supplementary material for: Understanding the Engagement and Interaction of Superusers and Regular Users in UK Respiratory Online Health Communities: Deep Learning–Based Sentiment Analysis
Source: J Med Internet Res. 2025 Feb 13;27:e56038. doi: 10.2196/56038 (PMC11888069; doi:10.2196/56038)
Supplement: Multimedia Appendix 1 [file jmir_v27i1e56038_app1.docx]

**Positive (rephrased)**:

AUK:

A user shared that they had returned home safely and were now resting, enjoying the comfort of having tea prepared for them. They wished everyone a pleasant evening.

BLF:

A user emphasized the importance of ensuring that everyone with COPD is referred to pulmonary rehabilitation, noting the significant positive impact it had on their condition. They highlighted the need to maintain regular exercise and activity even after completing the program. Sharing their own routine, they mentioned exercising about four times a week for 30 minutes, sometimes taking breaks by adopting a "do a bit, rest a bit, do a bit more" approach. They found this routine had improved their strength and ability to manage their condition and encouraged others to stay active.

**Negative (rephrased)**:

AUK:

A user described their experience with respiratory issues, sharing that they often dealt with coughing, wheezing, shortness of breath, and severe chest tightness. They reassured others facing similar struggles that they are not alone. The user recalled first experiencing breathing difficulties at age 13, which was initially attributed to bad hay fever and mild asthma. Their treatment at the time included a nasal spray and a month-long course of reliever tablets, though the tablets were discontinued, and the spray remained. At 19, after moving to a different county and changing GPs, they were again diagnosed with asthma and began using various inhalers. However, two years ago, they were informed it wasn’t asthma, though the cause remained unclear. After further visits, they were eventually diagnosed with Bronchorrhea (suspected spelling), a condition where the lungs produce excessive mucus, which they were told was just another symptom. The user shared that they are now being treated at their local hospital again as their chest issues have worsened. Unfortunately, they will not see a new consultant until May, leaving them in a difficult position in the meantime.

BLF:

A user shared that they had been feeling very low recently and came to the realization that they were experiencing depression. They described being irritable with colleagues and family members and having frequent emotional outbursts, which they recognized as classic symptoms. As a result, they were prescribed anti-depressants and given a two-week break from work. Reflecting on possible causes, they noted that significant changes at work, not always positive, had impacted their mental health. They also expressed frustration at the lack of understanding from others regarding long-term lung conditions such as bronchiectasis. They emphasized that these conditions are chronic and don’t improve, with good days and bad days being part of daily life. The user explained how their treatment regime dictates their routine, as they must carefully plan activities, such as going out in the evening, around their nebulized medications, salbutamol, colomycin, and the use of a smartvest. They pointed out that while others might work on their fitness to improve health, those with chronic lung conditions must constantly put in effort just to prevent their lungs from deteriorating further.

**Neutral (rephrased)**:

AUK:

A user shared that their 21-year-old daughter was advised by a doctor to take Seretide and Symbicort, along with Singulair at night and Bricanyl as needed. They mentioned that their daughter had used Symbicort before but never in combination with Seretide, and they were uncertain if it was standard practice to use both medications simultaneously. They expressed confusion, as they had previously understood it to be a case of using one or the other. Seeking clarity, they reached out to the community for advice and insights.

BLF:

A user asked whether everyone with COPD has had the opportunity to be seen by a consultant.
