# Supplementary material for: Understanding the Engagement and Interaction of Superusers and Regular Users in UK Respiratory Online Health Communities: Deep Learning–Based Sentiment Analysis
Source: J Med Internet Res. 2025 Feb 13;27:e56038. doi: 10.2196/56038 (PMC11888069; doi:10.2196/56038)
Supplement: Multimedia Appendix 2 [file jmir_v27i1e56038_app2.docx]

## Results with superusers identified by VoteRank


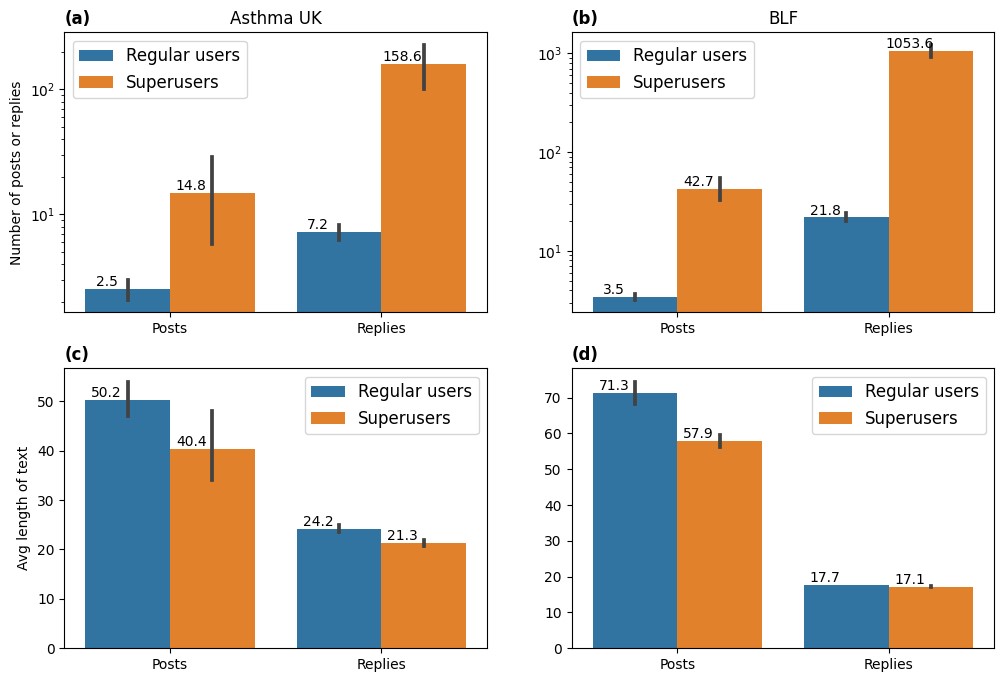


**Figure 10.** Comparisons of number and length of posts and replies written by superusers (identified by VoteRank) and regular users


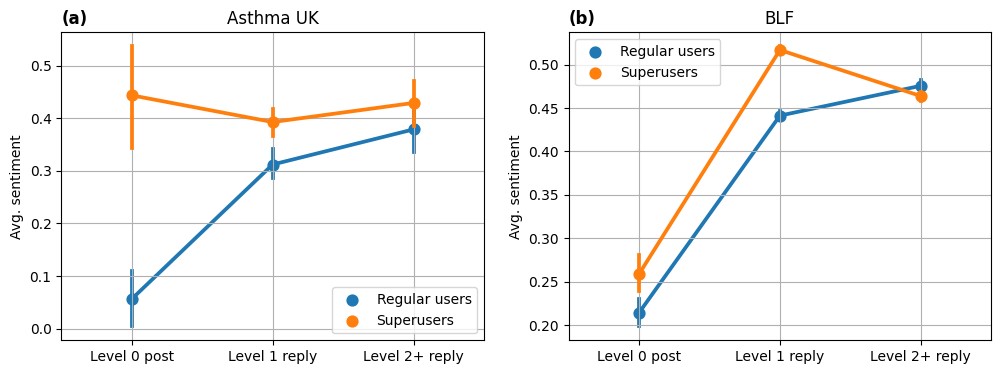


**Figure 11**. Sentiment in different actions (superusers identified by VoteRank). $\mathrm{AVS}_{v}^{0-\text{posts}\text{ }}$, $\mathrm{AVS}_{v}^{1-\text{replies}\text{ }}$and $\mathrm{AVS}_{v}^{2-\text{replies}\text{ }}$are computed for average sentiment in the corresponding categories.


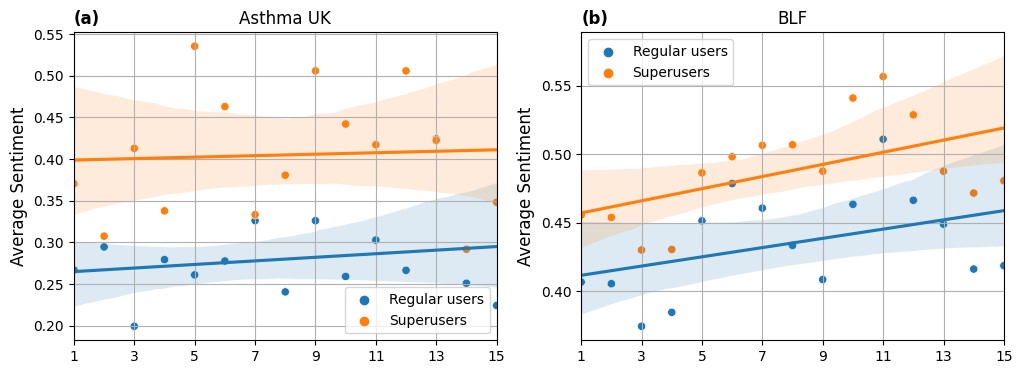


**Figure 12**. Trend of sentiment (superusers identified by VoteRank). All posts are sorted based on their publication time and regrouped into 15 *bins* with an equal volume. We computed the $\mathrm{AVS}_{v}^{\text{posts}\text{ }}$ in each bin.


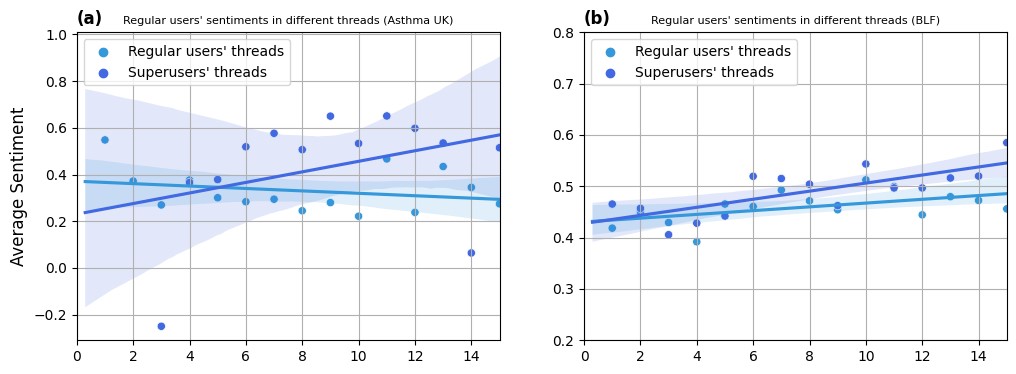


**Figure 13.** Sentiment trend in interactions between superusers (identified by VoteRank) and regular users. $\mathrm{AVS}_{U}^{\text{replies}}\left( T_{U} \right)$and $\mathrm{AVS}_{U}^{\text{replies}}\left( T_{S} \right)$are computed in each bin for points of regular users’ threads and superusers’ threads respectively.


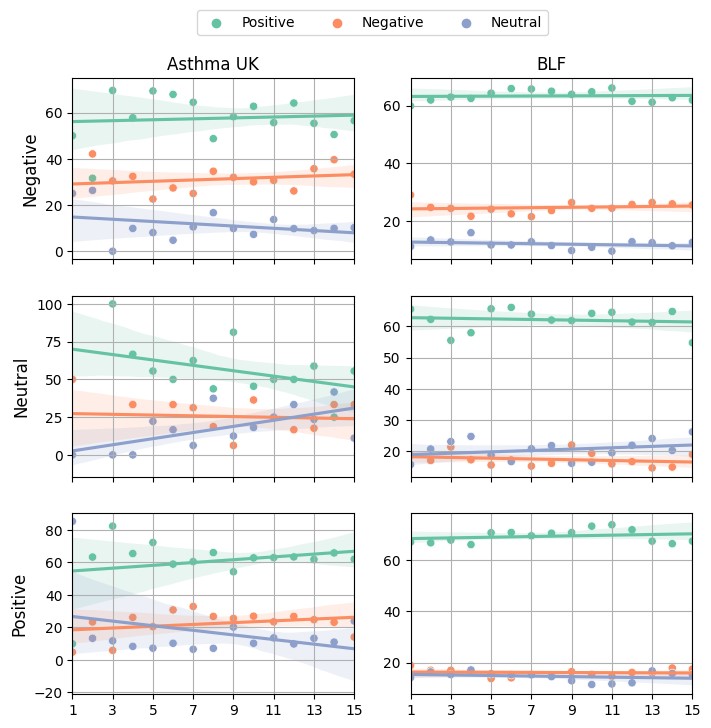


**Figure 14.** Sentiment trend in replies of superusers (identified by VoteRank) to regular users.

**Table 5**. Percentages of posts or replies with different sentiment and the average sentiment, indicated as AVS. Depending on the target, it refers to either $\mathrm{AVS}_{v}^{\text{posts}\text{ }}$, $\mathrm{AVS}_{v}^{0-\text{posts}\text{ }}$or $\mathrm{AVS}_{v}^{\text{replies}\text{ }}$ (superusers identified by VoteRank).

|  | AUK | |  | BLF | |
| --- | --- | --- | --- | --- | --- |
|  | Superusers | Regular users |  | Superusers | Regular users |
| **All posts** |  |  |  |  |  |
| Negative | $22.59\%$ | $30.70\%$ |  | $17.75\%$ | $20.98\%$ |
| Neutral | $14.32\%$ | $10.82\%$ |  | $15.58\%$ | $14.72\%$ |
| Positive | $63.09\%$ | $58.49\%$ |  | $66.66\%$ | $64.30\%$ |
| AVS | 0.405 | 0.278 |  | 0.489 | 0.433 |
| **level-0 posts** |  |  |  |  |  |
| Negative | $26.37\%$ | $43.17\%$ |  | $31.65\%$ | $33.32\%$ |
| Neutral | $2.89\%$ | $8.04\%$ |  | $10.83\%$ | $11.95\%$ |
| Positive | $70.74\%$ | $48.79\%$ |  | $57.52\%$ | $54.73\%$ |
| AVS | 0.444 | 0.056 |  | 0.259 | 0.214 |
| **Replies** |  |  |  |  |  |
| Negative | $22.37\%$ | $27.62\%$ |  | $17.28\%$ | $19.76\%$ |
| Neutral | $15.00\%$ | $11.50\%$ |  | $15.75\%$ | $14.99\%$ |
| Positive | $62.64\%$ | $60.88\%$ |  | $66.97\%$ | $65.25\%$ |
| AVS | 0.403 | 0.333 |  | 0.497 | 0.455 |

**Table 6**. Interactions between superusers (identified by VoteRank) and regular users. Depending on the target, AVS of posts and AVS of replies refer to either $\mathrm{AVS}_{S}^{0-\text{posts}}$ and $\mathrm{AVS}_{U}^{\text{replies}}\left( T_{S} \right)$, or $\mathrm{AVS}_{U}^{0-\text{posts}\text{ }}$ and $\mathrm{AVS}_{S}^{\text{replies}}\left( T_{U} \right)$. Posts without any replies are excluded in the calculation of AVS
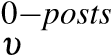
.

|  | Threads in AUK | |  | Threads in BLF | |
| --- | --- | --- | --- | --- | --- |
|  | Superusers' | Regular users' |  | Superusers' | Regular users' |
| Num. total threads | 311 | 1369 |  | 7053 | 13703 |
| Num. threads with replies | 245 | 855 |  | 5971 | 11631 |
| AVS of posts | 0.441 | 0.025 |  | 0.247 | 0.156 |
| AVS of replies | 0.502 | 0.358 |  | 0.488 | 0.479 |
| $L$ of posts | 36.237 | 46.163 |  | 68.836 | 42.487 |
| $L$ of replies | 15.546 | 24.858 |  | 15.770 | 19.658 |

**Table 7.** Summary of superusers’ threads and how regular users replied to them (superusers identified by VoteRank). $\mathrm{AVS}_{U}^{\text{replies}}\left( T_{S}^{+} \right)$, $\mathrm{AVS}_{U}^{\text{replies}}\left( T_{S}^{-} \right)$, and $\mathrm{AVS}_{U}^{\text{replies}}\left( T_{S}^{=} \right)$, are computed for AVS in corresponding columns.

|  | Sentiment of superusers (AUK) | | |  | Sentiment of superusers (BLF) | | |
| --- | --- | --- | --- | --- | --- | --- | --- |
|  | Negative | Neutral | Positive |  | Negative | Neutral | Positive |
| Number of posts | 82 | 9 | 220 |  | 2232 | 764 | 4057 |
| Pct. reply | 79.27% | 77.78% | 78.64% |  | 87.23% | 78.53% | 84.40% |
| AVS of replies | 0.26 | 0.76 | 0.56 |  | 0.37 | 0.46 | 0.51 |
| $L$ of replies | 20.61 | 15.14 | 16.78 |  | 18.57 | 12.56 | 15.74 |

**Table 8**. Percentage of replies with different sentiment and average sentiment based on the sentiment in the *level-0 post*s in each thread written by regular users (superusers identified by VoteRank). $\mathrm{AVS}_{S}^{\text{replies}}\left( T_{U}^{+} \right)$, $\mathrm{AVS}_{S}^{\text{replies}}\left( T_{U}^{-} \right)$, and $\mathrm{AVS}_{S}^{\text{replies}}\left( T_{U}^{=} \right)$are computed for AVS in corresponding columns. Regular users’ replies to other regular users’ *level-0 posts* are used as a baseline and compared with AVSs of superusers.

|  | Sentiment of regular users (AUK) | | |  | Sentiment of regular users (BLF) | | |
| --- | --- | --- | --- | --- | --- | --- | --- |
|  | Negative | Neutral | Positive |  | Negative | Neutral | Positive |
| **Regular users' replies** |  |  |  |  |  |  |  |
| Negative | 40.09% | 19.08% | 31.50% |  | 29.69% | 21.01% | 19.30% |
| Neutral | 9.96% | 33.59% | 12.77% |  | 11.91% | 21.83% | 14.13% |
| Positive | 49.94% | 47.33% | 55.73% |  | 58.40% | 57.16% | 66.57% |
| Total | 100% | 100% | 100% |  | 100% | 100% | 100% |
| AVS | 0.098 | 0.282 | 0.242 |  | 0.287 | 0.361 | 0.473 |
| **Superusers' replies** |  |  |  |  |  |  |  |
| Negative | 32.18% | 25.00% | 24.90% |  | 25.19% | 16.91% | 15.40% |
| Neutral | 7.80% | 18.10% | 11.37% |  | 10.08% | 19.87% | 12.54% |
| Positive | 60.02% | 56.90% | 63.73% |  | 64.73% | 63.22% | 72.06% |
| Total | 100% | 100% | 100% |  | 100% | 100% | 100% |
| AVS | 0.098 | 0.282 | 0.242 |  | 0.287 | 0.361 | 0.473 |
